# HLA Immune Function Genes in Autism

**DOI:** 10.1155/2012/959073

**Published:** 2012-02-15

**Authors:** Anthony R. Torres, Jonna B. Westover, Allen J. Rosenspire

**Affiliations:** ^1^Center for Persons with Disabilities, Utah State University, 6804 Old Main Hill, Logan, UT 84322, USA; ^2^Department of Immunology and Microbiology, School of Medicine, Wayne State University, 221 Lande Building, Detroit, MI 48201, USA

## Abstract

The human leukocyte antigen (HLA) genes on chromosome 6 are instrumental in many innate and adaptive immune responses. The HLA genes/haplotypes can also be involved in immune dysfunction and autoimmune diseases. It is now becoming apparent that many of the non-antigen-presenting HLA genes make significant contributions to autoimmune diseases. Interestingly, it has been reported that autism subjects often have associations with HLA genes/haplotypes, suggesting an underlying dysregulation of the immune system mediated by HLA genes. Genetic studies have only succeeded in identifying autism-causing genes in a small number of subjects suggesting that the genome has not been adequately interrogated. Close examination of the HLA region in autism has been relatively ignored, largely due to extraordinary genetic complexity. It is our proposition that genetic polymorphisms in the HLA region, especially in the non-antigen-presenting regions, may be important in the etiology of autism in certain subjects.

## 1. Autism

Leo Kanner first described autism in 1943 [1] after finding 11 children with common symptoms of obsessiveness, stereotypy, and echolalia at Johns Hopkins University. Autism remained an esoteric disorder for several decades until physicians and parents connected these symptoms with an increasing number of patients. It is important to note that the diagnostic criteria have been modified over the years to include a broader category of symptoms, thus increasing the number of children diagnosed with the disorder, now referred to as Autism Spectrum Disorder (ASD) [2]. Currently, the Centers for Disease Control and Prevention (CDC) states that the incidence of ASD is 1 out of 110 children in the United States [3]. The severity of ASD varies greatly with the most severe forms, much like Kanner autism, displaying language regression, seizures, and lower IQ. Altevogt et al. [4] have suggested that autism, or more properly ASD, is not a single disorder, but a collection of similar disorders each with different characteristics and perhaps etiologies.

Even after several decades of research, there is much debate around the world on the etiology of ASD. It is clear that ASD results from abnormal brain development in either the prenatal period or infancy stage of life. Exposure to mercury, maternal viral infections, autoimmune disorders, and the inheritance of certain gene combinations have been implicated in the etiology. Unfortunately, none of these areas have given clear answers as to the etiology. Fortunately, psychologists have made significant strides in treating children and it appears the earlier behavioral treatment starts, the better the outcome. Nevertheless, medical researchers continue to search for the cause(s) of ASD. This paper discusses a possible role for the immune system, and in particular immune function genes in the human leukocyte antigen region (HLA), as a research area that should be more closely investigated.

## 2. Infections

One of the first areas of interest in the 1960s and 1970s was the search for an infectious agent that might be involved in the etiology of ASD [5]. During this time, there were many case reports in the literature that suggested an association between congenital rubella infection and resultant autistic behaviors. However, after decades of research no definite role for infectious agents in autism etiology has been confirmed. On the other hand, these endeavors have led to observations that perhaps the immune system was involved in autism, and evidence continues to mount that immune abnormalities are indeed associated with ASD.

## 3. Familial Studies

Studies indicating familial clustering and the increases of ASD in twins have been interpreted by many as an indication of genetic predisposition. Twin studies show the concordance rates of monozygotic twins at 36–96%, whereas dizygotic twins are 0–24% concordant, resulting in an estimated heritability of autism at >90% [6–8]. Additionally, family studies have shown autism to have familial aggregation with 3–8% of subsequently born siblings either being autistic or showing some form of pervasive developmental disorder (PDD) [9, 10]. This is a 3- to 8-fold increase in risk for siblings over the general population. A more recent study gave an estimate of 18.7% sibling recurrence risk for ASD, a 20-fold increase over the general population [11]. It is important to note, however, that family data should be looked at with great caution, as individuals living in the same household will have similar exposures to microorganisms and environmental chemicals. Taking this into account, a recent paper based on data from twin pairs estimates the genetic heritability for ASD to be 14–67% [12]. However, this idea is somewhat controversial as many in the research community continue to feel that the results from family studies are indicative of a strong genetic etiology [13, 14].

## 4. Genetics

Many of the early genetic studies involved the examination of microsatellites throughout the human genome in an attempt to find genomic regions that would associate with autism. Overall, this approach was not very fruitful and researchers quickly started to examine single-nucleotide polymorphisms (SNPs) as techniques advanced [15]. With this approach many researchers proposed that multiple candidate genes were associated with ASD. Unfortunately, studies proposing candidate genes were often contradictory and proved to be unreliable [15]. One of the most interesting genetic findings in ASD is the association of autism with the MET receptor tyrosine kinase gene located on chromosome 7q31 as MET signaling participates in gastrointestinal repair, immune function, neocortical, and cerebellar growth [16]. It is important to mention that the autism MET associations have been replicated by other research groups [17]. This autism association showed a relative risk of 2.27 which is much lower than the relative risk for HLA gene/allele associations discussed below.

It is very reasonable to believe that deletions or duplications of genetic regions, which can cause lower or higher levels of gene expression, could produce pathological phenotypes. Consequently, newer approaches examining copy number variation (CNV) and microarray analyses of 500,000 SNPs or more have been in vogue for several years in the study of ASD. These newer approaches have identified CNV mutation differences in genes involved in neuronal cell adhesion and ubiquitin degradation as being associated with ASD [18]; however, these results have yet to be replicated by other researchers. 

 The neurexin-1 gene has been associated with a variety of developmental disorders including ASD [19]. The neurexin-1 gene (2p16.3) is one of the largest genes in the human genome with 24 exons in 1.1 Mb. With two independent promotors there can be over 1,000 neurexin isoforms generated from the 24 exons in different cells or tissue. Another gene of interest is the contactin-associated protein-like 2 (CNTNAP2) gene that was shown to be associated in Old-Order Amish subjects with intractable epilepsy and ASD [20]. Three other groups have now confirmed the involvement of CNTNAP2 in ASD [21–23].

Both the neurexin-1 and the CNTNAP2 genes are involved in synaptic function. Although these approaches have shown a strong association of certain genes with ASD, only a small percentage of subjects with ASD have these mutations. For example, the neurexin-1gene is found in only about 0.5% of autism cases and 0.2% of controls. The 90% missing inheritance may be largely due to marked genetic heterogeneity, suggesting that different ASD phenotypes should be examined separately [24–26]. Recent genetic research has also associated numerous immune function genes with autism [27–30]. A large study that examined SNP data from several genomewide scans on 3,130 subjects with schizophrenia found that the 5 most significant SNP markers are found across the HLA region [31]. It appears that some of missing inheritance, at least in schizophrenia, was uncovered in the HLA region and we suggest a similar finding will be confirmed/uncovered in autism.

## 5. Immune Abnormalities in Autism

It is no surprise to see immune gene associations in ASD, as numerous researchers have reported immune abnormalities in autism for over 20 years. It has become increasingly obvious that inflammatory processes are associated with autism. Blood levels of the inflammatory cytokines IL-6, INF-*γ*, and TNF-*α* were shown to be elevated in autistic individuals compared to controls [32, 33]. Later, seminal work by Vargas et al. [34] utilized direct morphological analysis and immunohistologic techniques to show an active neuroinflammatory process in the cerebral cortex, white matter, and in particular the cerebellum of ASD patients that was dependent on activation of microglia and astroglia. Cytokine profiling demonstrated that neuroinflammation was accompanied by upregulation of the macrophage chemoattractant protein (MCP-1) and TGF-beta in brain tissue, and that MCP-1 was also upregulated in cerebral spinal fluid [34]. More recent work has directly demonstrated that aside from blood, IL-6, TNF-*α*, and INF-*γ* are elevated in ASD brains, along with the other inflammatory cytokines GM-CSF and IL-8 [35]. Most recently, it has been reported that the important inflammatory-associated transcription factor, nuclear factor kappa-light-chain-enhancer of activated B cells (NF-*κ*B) is upregulated in both blood [36] and brain tissue [37] of autistic individuals. Other immune abnormalities such as autoantibodies started to be reported in 1993 [38] (Table 1).

Autoantibodies to myelin basic protein have been noted by at least a couple of researchers [38, 52]. An increase in autoantibody reactivity has been reported against other brain proteins in ASD including nerve growth factor [53], brain endothelium [54], cerebellar proteins [40], and serotonin 5-HT receptors [55] and transglutaminase-2, a protein important in synaptic stabilization [39]. Croonenberghs et al. [56] noted a significant increase in gamma globulin especially of the IgG2 and IgG4 subclasses in children with autism over a control population. Autoantibodies to several uncharacterized brain-specific proteins have been reported in the plasma of autistic individuals. In particular, western blot analysis has shown the presence of IgG autoantibodies targeting a protein of approximately 52 kDA located in the hypothalamus and thalamus of adult brain [45]. Other autoantibodies targeting three brain proteins of 43–48 kDA located in the hypothalamus have also been observed in the serum of autistic individuals [45]. Autoantibodies targeting cerebellar proteins of 45 and 62 kDA have been associated with ASD [40]. These autoantibodies may be specific to cerebellar Golgi cells, which are GABAergic interneurons [41]. Autoantibodies reactive to human brain proteins in the 36–39 and 61 kDA range have also been found in the sera of mothers of autistic children. [57, 58].

It is important to note that while autoantibodies associated with ASD may be biomarkers, they may not necessarily be pathologic in and of themselves. Central tolerance refers to the process whereby immature lymphocytes are negatively selected based on the ability of their antigen receptors (the B cell receptor or BCR for B cells and the T-cell receptor or TCR for T cells) to recognize self-antigens [59–61]. Until fairly recently, it was believed that most immature lymphocytes recognizing self-antigens (autoimmune repertoire) were normally neutralized by virtue of central tolerance before they could mature, and that peripheral tolerance would insure the removal of any self-reactive lymphocyte escaping central tolerance. Peripheral tolerance refers to the process whereby mature self-reactive lymphocytes which have escaped central tolerance are eliminated, largely by CD95-mediated apoptosis [62]. Thus responses to foreign antigens were viewed as normal, while anti-self responses were considered necessarily pathologic. However, with the realization that many self-reactive lymphocytes survive central and peripheral tolerance, this view has had to be modified [49]. Limited immune responses to self-antigens (autoimmunity) are now understood to be normal and not necessarily pathologic [63].

Upon stimulation of the system with a pathogen, cognate lymphocyte clones representative of the antiforeign repertoire normally expand and mature, providing a protective immune response. On the other hand, it is the expansion and maturation of those clones representative of the autoimmune repertoire that leads to autoimmune disease. In other words, the developing immune system can be characterized as balancing production between antiforeign (protective) and antiself (autoimmune) repertoires. While a beneficial function of naturally occurring, low level autoimmune antibodies, also referred to as natural antibodies (NAs), remains a matter of debate, it appears as if the repertoire of NA is reflective of the susceptibility to develop specific autoimmune diseases [64, 65]. Because central tolerance of T cells depends to a large extent upon the strength of the TCR interaction with an autoantigen or an HLA class I or II complex [59], the NA repertoire will to a large extent depend upon the HLA haplotype, with some haplotypes favoring autoantibodies targeting one antigen over another. For instance, in the case of ASD we have found an association with low level antibody responses to tissue transglutaminase, and that this response appears linked to the HLA-DR3/DQ2 and DR7/DQ2 haplotypes [39]. It is likely that the other autoantibodies noted above as being associated with ASD may be linked to different haplotypes.

Aside from autoantibodies and altered cytokine levels there appear to be other immune abnormalities associated with ASD. It was noted that there were decreased numbers of T-lymphocytes and an altered ratio of suppressor T-lymphocytes to helper T-lymphocytes [66, 67] and altered T-lymphocytes responses in children with autism [68]. Warren et al. [69] reported that subjects with autism had reduced NK cell killing in the standard K562 target cell cytotoxicity assay. This observation of decreased NK cell killing has been repeated by at least a couple of other research teams [28, 70]. One research group [71] observed that monocyte counts and neopterin levels were increased in autistic children compared to gender and age-matched healthy controls suggesting that the immune system was overactivated in the ASD group. Another elegant set of experiments involved the stimulation of cultured monocytes with several toll-like-receptor (TLR) ligands. The monocytes from subjects with ASD had significant increases or decreases in proinflammatory cytokines depending on the particular TLR ligand added to the cell culture media [72].

 One large-scale study found that the frequency of autoimmune disorders in the families with autistic children was found to be higher than in control subjects, especially mothers of autistic children [73]. Another group demonstrated that autoimmune diseases were increased significantly in families with ASD compared with those of healthy control subjects [74], suggesting a link between the disorders. Croen et al. [75] showed that maternal psoriasis diagnosed around the time of pregnancy is significantly associated with a subsequent diagnosis of autism in the child. Additionally, they showed a 2-fold increase in risk for a child having ASD if the mother was diagnosed and with asthma or allergies during pregnancy. An association between a family history of type 1 diabetes mellitus (T1DM) and infantile autism as well as a significant association between maternal histories of either rheumatoid arthritis (RA) or celiac disease and ASDs was noted by Atladóttir et al. [76].

## 6. Autism HLA Genetics

**Figure 1:**
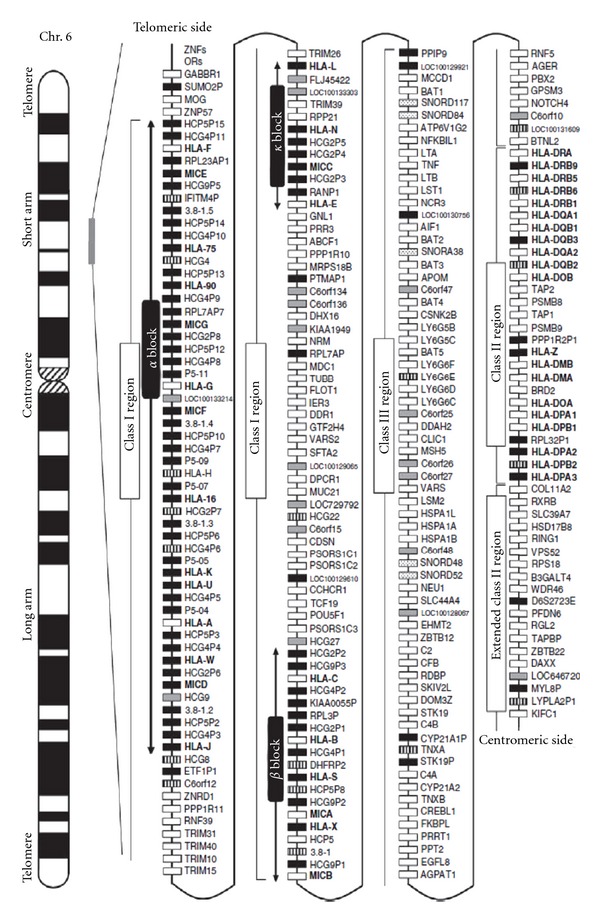
Gene map of the human leukocyte antigen (HLA) region. The major histocompatibility complex (MHC) gene map corresponds to the genomic coordinates of 29677984 (GABBR I) to 33485635 (KIFC1) in the human genome build 36.3 of the National Center for Biotechnology Information (NCBI) map viewer. The regions separated by arrows show the HLA subregions such as extended class I, classical class I, class III, classical class II, and extended class II regions from telomere (left and top side) to centromere (right and bottom side). White, gray, striped and black boxes show expressed genes, gene candidates, noncoding genes and pseudogenes, respectively. The location of the alpha, beta, and kappa blocks containing the cluster of duplicated HLA class I genes in the class I region are indicated. (Reprinted with permission from the *Journal of Human Genetics* [87].)

HLA is the name for the major histocompatibility complex (MHC) in humans and HLA and MHC are often used interchangeably in the literature. The HLA region on chromosome 6p21 (about 4 × 10^6^ bp) is of major interest in basic research as well as medicine as genes/proteins in this region are involved in many biological processes such as histocompatibility, inflammation, ligands for immune cell receptors, and the complement cascade. The HLA region has 20 typical HLA genes and 112 nontypical HLA genes (Table 2) that are inherited together as frozen blocks of DNA called ancestral or extended haplotypes. Complete DNA sequences have been published for 8 of the more common ancestral haplotypes in an effort to expedite basic and disease research [77]. It should be mentioned that smaller haplotypes can also be constructed for genes that are linked. HLA genes also play a role in reproduction, pregnancy maintenance, mate selection, and even kin recognition [78, 79] and have been associated with over 100 diseases/disorders including autism. The proteins encoded by HLA genes are ligands, receptors, cytokines, signaling factors, heat shock proteins, transcription regulators, and so forth. Current research is increasingly demonstrating a role for HLA proteins in neural cell interactions, synaptic function, cerebral hemispheric specialization, central nervous system (CNS) development [80–84], and even neurological disorders [85]. The genes of the HLA region are shown in Figure 1. Shiina et al. [86, 87] have published two excellent reviews on the HLA super-locus. Not only is there extraordinary complexity in the HLA genes, there are extensive haplotype-related transcriptional differences [88]. It has been shown that genetic mechanisms outside of the non-antigen-binding HLA genes in the ancestral haplotype 8.1 (also referred to as COX) are associated with susceptibility to many autoimmune diseases [89]. It is our proposition that HLA genes/proteins should be more carefully examined due to increasing evidence of autoimmune type associations in autism.

## 7. HLA Associations in ASD

 It was suggested over 30 years ago by Stubbs and Magenis [90] that the HLA region might be important in autism. Warren et al. [91] first reported that the HLA ancestral haplotype 44.1 (B44-SC30-DR4) was associated with autism with a relative risk of 7.9. That result was confirmed in a separate case/control population [92]. Interestingly, the individual components of AH 44.1 (A2-B44-SC30-DR4) include a deleted C4B gene and DR*β*1*0401, both of which have been shown independently to be significantly associated with ASD [93, 94]. Examination with different genetic markers than those used by Warren suggested that certain HLA haplotypes are associated with autism in Sardinian and Italian families [95, 96].

Warren et al. [97] reported that the shared epitope-binding pocket (DR*β*1*0401, *0404, and *0101) in the third hypervariable region of DR*β*1 has a strong association with autism. A relative risk of 19.8 for autism was reported for subjects with one of the two extended HLA haplotypes. Both of these haplotypes have many allelic similarities especially the DR*β*1*0401.

AH 44.1 (HLA-A2, Cw5, B44, Bf*S, C2*C, C4A3, C4BQ0, DR*β*1*0401, DQB1*0301).

AH 62.1 (HLA-A2, Cw3, B15, Bf*S, C2*C, C4A3, C4B3, DR*β*1*0401, DQB1*0302).

The shared epitope has been associated with several autoimmune diseases such as rheumatoid arthritis, psoriatic arthritis, and systemic lupus erythematosus [98]. Torres et al. [93] confirmed the association of the HLA-DR4 allele and also found that the DR13 and DR14 alleles occurred less often in subjects with autism, suggesting a possible protective mechanism. Interestingly, the DR13 allele was inherited less frequently than expected from the mothers. Associations with autism and the DR4 allele have since been confirmed in three additional research groups. Lee et al. [99] demonstrated that boys with autism and their mothers had a significantly higher frequency of DR4 than normal control subjects (odds ratios 4.20 and 5.54, resp.), suggesting that a maternal-fetal immune interaction could be involved in autism. Johnson et al. [100] reported significant transmission disequilibrium for HLA-DR4 (odds ratio 4.67) from maternal grandparents to mothers of children with autism which also suggests a maternal-fetal interaction for HLA-DR4. It has been recently shown in Han Chinese that the HLA-DR*β*1 allele frequencies including DR4 are different in subjects with autism versus control subjects [101].

It was reported 20 years ago that subjects with autism had a significant increase in the C4B null allele (C4B gene deletion) compared to control subjects [91]. After this observation, it was also noted that subjects with autism had a significant deficiency in the plasma C4B protein [102]. These initial findings of an increase in the deletion of the C4B gene was supported by examining a new population of subjects with autism [94]. Mostafa and Shehab [103] have recently reported a significant increase in the deletion of the C4B gene in the Egyptian population. They also reported an increased risk when there was a family history of autoimmune diseases in the autism population. Descriptions of several non-antigen-binding HLA genes that are associated with autoimmune diseases are discussed below and listed in Table 3.

## 8. Non-Antigen-Binding HLA Genes in Class I

Although MICA/MICB genes are structurally similar to classical antigen-binding HLA class I genes, they encode proteins that interact with different T-cell receptors in response to stress as infection or neoplastic transformation. The two genes are located on the centromeric end of the class I region near HLA-B at the border of the class III region (Figure 1) [87]. MICA/MICB proteins are ligands for NKG2D receptors on NK cell and gamma/delta T-cell receptors. Adaptive immunity involves three lymphocyte populations (B cells, alpha/beta T cells, and gamma/delta T cells). Gamma/delta T cells represent a small population of T cells that possess a T-cell receptor that is distinct from the typical alpha/beta T cell. They are concentrated in the intestinal mucosa and appear to have a prominent role in recognizing small bacterial phosphoantigens and undefined antigens presented by MICA/MICB proteins. Gamma/delta T cells have potent cytotoxic activity and have been considered a link between innate and adaptive immunity. Polymorphisms in MICA/MICB genes have been associated with T1DM, AD, and SLE independent of DR*β*1 alleles. There are several genes associated with psoriasis (PSORSI locus). It is unclear how the risk is spread among these genes as they are closely linked. Two genes RNF39 and TRIM39 in the class I region have been associated with Behçet's disease.

## 9. Non-Antigen-Binding HLA Genes in Class III

Tumor necrosis factor-alpha (TNF-*α*) is a proinflammatory, multifunctional cytokine that plays important roles in cell physiology. It is synthesized in numerous cells including macrophages, NK cells, T cells, mast cells, osteoblasts, granulocytes, smooth muscle cells, fibroblasts, and keratinocytes. In the CNS, TNF-*α* is made in microglia cells, astrocytes, and neurons [141]. In the normal state, TNF-*α* drives acute and chronic inflammatory responses that leads to the removal of injurious stimuli and the restoration of homeostasis. TNF-*α* is necessary for neural cell differentiation and neuron maturation and there is evidence that it is critical in the normal brain for proper synaptic function.

There is extensive research that implicates TNF-*α* as a key mediator in disease progression, inflammation, blood-brain-barrier deterioration, and even cell death [142]. Elevated levels are present in numerous neurological disorders including multiple sclerosis, Alzheimer's disease, Parkinson's disease, ischemia, traumatic brain injury, and as mentioned above, ASD [141]. It is unclear if TNF-*α* contributes to the disease state or the higher concentrations limit neuronal injury. There are three adjacent genes: lymphotoxin alpha and beta (LTA and LTB) and leukocyte-specific transcript-1 (LST1) in the TNF-*α* block. LTA and LTB are proinflammatory cytokines like TNF-*α*, and LST1 plays a role in inflammatory and infectious diseases. It has been suggested that vaccine-induced immunity changes with certain haplotypes in these genes [143]. Two other genes important in immune function are adjacent to the TNF-*α* block. Nuclear factor of kappa light polypeptide gene enhancer in B-cells inhibitor-like 1(NF-*κ*BIL1) on the telomeric side of TNA-*α* is an inhibitor of NF-*κ*B, an important pleiotropic immune system transcription factor that is upregulated in ASD [144, 145]. In addition, NF-*κ*BIL1 has been associated with several autoimmune diseases (Table 3). The natural cytotoxicity trigger receptor three (NCR3) gene on the centromeric side encodes proteins that are ligands for activating receptors on NK cells that recognize tumor cells [146]. Typical HLA-B and -C proteins are also ligands for KIR receptors on NK cells.

There are numerous heat shock proteins (HSPs), also referred to as chaperones, that assist the folding of newly synthesized proteins as well as those that have been unfolded or denatured [147]. HSPs are also important in cell protective functions and in inhibiting the apoptosis cascade. They are named by their molecular weights (HSP100, HSP90, HSP70, HSP60, and smaller HSPs). Although the proteins appear in normal cellular functions, they are induced to higher levels in trauma, epilepsy, neurodegenerative diseases, and other injuries. An underlying feature among AD, Parkinson's disease, spinocerebellar ataxia, and other neurodegenerative diseases is the accumulation of misfolded proteins and HSPs are being studied because of their role in folding and refolding proteins [147]. There is intense interest in HSPs as they appear to protect neurons [148] and one must remember that postmitotic neurons are unable to dilute misfolded or aggregated proteins through division.

There are three HSP70 genes (HSPA1L, HSPA1A, and HSPA1B) in the class III HLA region that are adjacent but separate genes. HSP70 proteins have been demonstrated to stimulate IL-6 and TNF-*α* production, activate microglial cells, and stimulate phagocytosis [148]. HSP70 proteins are also important in autoimmunity by enhancing antigen presentation in both HLA class I and HLA class II systems [147, 149]. Peptides that are associated with HSP70 at the time of T-cell presentation have been shown to be more antigenic and therefore involved in autoimmunity [149, 150].

Another gene that is independently associated with autoimmune diseases outside of non-antigen-binding HLA alleles is allograft inflammatory factor-1 (AIF1). The SNP rs2269475 C > T in AIF1 has been associated with RA. The T allele was significantly higher in the RA patients and there was no significant linkage disequilibria between the AIF1 SNP and DR*β*1 alleles. Anticyclic citrullinated peptide antibodies commonly used to monitor RA were significantly increased in carriers with the T allele [151] but not the C allele.

## 10. Non-Antigen-Binding HLA Genes in Class II

Although HLA-DM and -DQ proteins have structures like classical antigen-binding HLA proteins, they work in the cytoplasm and not at the cell surface antigen-presenting HLA proteins. DM stabilizes and edits the peptide repertoire presented by DQ proteins by catalyzing CLIP release. The associations of DQ2 with T1DM and celiac disease have been known for several decades; however, the biochemistry behind these associations has not been elucidated. It is now known that DQ2 is a poor substrate for DM and it has been proposed that antigen presentation in the thymus and periphery can be affected by impaired DQ-DM interactions so as to promote autoimmune disease [152]. HLA-DO is another nonclassical class II HLA protein that is involved in the loading of peptides to HLA-DR proteins by modulating the function of HLA-DM [153].

There are two genes in the class II region that encode proteins involved in the transport of antigen from the cytoplasm to the endoplasmic reticulum for binding to class I HLA proteins: transporter associated with antigen-processing 1 and 2 (TAP1 and TAP2). Several research groups have reported associations of TAP2 genes with SLE independent of classical HLA alleles [154]. This interaction is very interesting as it means that class II genes can affect class I antigen binding.

There are two other genes in the extended class II region that have been associated with autoimmune diseases (Table 3). Hydroxysteroid 17-beta dehydrogenase 8 (HSD17*β*8) is important in regulating the concentrations of active estrogens and androgens and high levels of estrogen are well known to be associated with SLE and other autoimmune diseases. The second gene, death-associated protein 6 (DAXX), is a very important protein that interacts with a variety of proteins in the nucleus and cytoplasm. Perhaps most importantly, it interacts with the death receptor Fas. Engstrom et al. [155] published a paper describing decreased expression of Fas on CD4 + T lymphocytes but higher serum levels of soluble Fas in ASD.

## 11. Summary

There is mounting evidence that the immune system plays a role in the pathogenesis of ASD in certain individuals. This evidence comes from several research areas including an increase in proinflammatory cytokines in blood and brain, autoantibodies to numerous antigens, and HLA associations.

Autism HLA associations have been observed across the entire HLA region. For example, in the class I region HLA-A2 has been associated with autism by at least two research groups [156, 157]. In the class II region several researchers have reported autism associations with the DR*β*1*04 allele [93, 99, 100]. Strong associations also appear in the class III region where the C4B null allele has been associated with autism with relative risks of 4.3 [94] and 4.6 [97], and an odds ratio of 6.3 [103]. The HLA-associated risk is the highest for autism (19.8) when combining two ancestral haplotypes (44.1 and 62.1). Both of these haplotypes have HLA-A2 and DR*β*1*0401 as well as other genetic similarities; however, these two alleles cannot account for all of the 19.8 risk. Compared to other genetic associations with autism, the HLA associations may be more important than realized, as they have the highest genetically associated risk, that we are aware of for autism. For example the MET gene, one of the most studies genetic regions in autism, has a relative risk of 2.27 [17].

It is our premise that some of the autism missing inheritance may be hidden in the HLA region, both in classical HLA alleles and nonclassical HLA genes, as seen in schizophrenia [31]. For example, the HLA class III region contains clusters of genes such as TNF-*α*, HSP70, C4A/C4B, and NF-*κ*BIL1 that are seminal in cellular function and are also associated with numerous autoimmune diseases (Table 3).

## Figures and Tables

**Table 1:** A list of proteins against which autoantibodies have been found in the serum of subjects with autism.

Protein	Reference
Transglutaminase 2	[39]
45 and 62 kDa proteins in cerebellum	[40, 41]
Voltage dependent anion channel (VDAC)	[42]
Hexokinase-1	[42]
Mitochondrial protein	[43]
Antimitochondrial DNA auto-antibodies	[43]
Nuclear proteins	[44]
52 kDa protein in hypothalamus and thalamus	[45]
43–48 kDa protein in the hypothalamus	[45]
Folate receptor	[46]
Brain-derived neuro trophic factor	[47]
HSP90	[48]
Myelin basic protein (MBP)	[38, 49]
Myelin-associated glycoprotein (MAG)	[50]
Myelin oligodendrocyte glycoprotein (MOG)	[50]
Neuron-axon filament protein	[51]
Glial fibrillary acidic protein	[51]

**Table 2:** Genes and alleles in the HLA region.

HLA genes	Number	HLA alleles	Number
HLA class I genes	6	HLA class I alleles	2215
HLA class II genes	12	HLA class II alleles	986
HLA class I-like genes	2	HLA class I-like alleles	94
Non-HLA genes	112		

Total genes	132		

HLA class I genes	A/B/C/E/F/G		
HLA class I-like genes	MICA/MICB		
HLA class II genes	DRA/DRB/DQ/DP/DM/DO		

**Table 3:** Non-classical HLA genes associated with autoimmune diseases.

	Gene	HGNC gene number	Autoimmune disease	References
Extended class I	OR2H2	8253	SLE	[104]

Class I	RNF39	18064	Behçet's disease	[105]
	TRIM39	10065	Behçet's disease	[105]
	PSORS1 locus	9573	Systemic sclerosis, Psoriasis	[106–111]
	MICA	7090	T1DM, AD, SLE	[112–114]
	MICB	7091	SLE	[104]

Class III	BAT1–BAT5	13917–21	Alzheimer's, AIDS	[115, 116]
	NFKBIL1	7800	Sjögren's syndrome, SLE, RA	[117, 118]
	TNF Block	11892	Alzheimer's, Psoriasis, Autoimmune hepatitis, Sarcoidosis	[115, 119–121]
	AIF1	352	T1DM	[122]
	HSP70 genes	5232–4	MS	[123]
	Complement genes	1248, 1324, 1323	SLE, myasthenia gravis, T1DM	[124–126]
	SKIV2L	10898	SLE	[127]
	ATF6B (CREBL1)	2349	SLE	[104]
	NOTCH4	7884	Systemic sclerosis	[128]
	C6orf10	13922	SLE	[104]
	BTNL2	1142	Ulcerative colitis, Sarcoidosis	[129, 130]

Class II	TAP2	44	Psoriasis	[131]
	PSMB8	9545	Hypersensitivity pneumonitis	[132]
			Psoriasis	[133]
	TAP1	43	Vitiligo	[134]
	PSMB9	9546	Psoriasis, Vitiligo	[133, 134]
	HLA-DM	4934, 4935	Psoriasis, Antiphospholipid auto-antibodies, RA, SLE, T1DM	[131, 135–138]
	HLA-DO	4936, 4937	common variable immunodeficiency	[139]

Class II Extended	HSD17B8	3554	SLE	[104]
	DAXX	2681	MS	[140]

Abbreviations: (HGNC) HUGO Gene Nomenclature Committee (http://www.genenames.org/); (SLE) systemic lupus erythematosus; (AIDS) acquired immune deficiency syndrome; (T1DM) type 1 diabetes mellitus; (AD) Addison's disease; (RA) rheumatoid arthritis; (MS) multiple sclerosis, PSORS1 psoriasis locus genes (CDSN, PSORS1C1, PSORS1C2, CCHCR1, POU5F1, PSORS1C3), TNF Block genes (LTA, TNF, LTB, LST1), HSP70 genes (HSPA1L, HSPA1A, HSPA1B), Complement genes (C2, C4B, C4A).
